# SLC16A1 Activates the STAT3/SLC7A11 Pathway to Mediate Ferroptosis Resistance and Tumor Progression in Head and Neck Squamous Cell Carcinoma

**DOI:** 10.32604/or.2026.077171

**Published:** 2026-04-22

**Authors:** Chunhui Tian, Weipin Xie, Wen Li, Huaiyu Gu, Xuebao Liu, Busheng Tong, Yehai Liu, Huaiyuan Zong

**Affiliations:** 1 Department of Otorhinolaryngology Head and Neck Surgery, The First Affiliated Hospital of Anhui Medical University, Hefei, China; 2 Department of Otorhinolaryngology Head and Neck Surgery, Suzhou Hospital Affiliated of Anhui Medical University, Suzhou, China; 3 Department of Biochemistry & Molecular Biology, School of Basic Medicine, Anhui Medical University, Hefei, China

**Keywords:** Head and neck squamous cell carcinoma (HNSCC), solute carrier family 16 member 1 (SLC16A1), signal transducer and activator of transcription 3 (STAT3), solute carrier family 7 member 11 (SLC7A11), ferroptosis, tumor growth

## Abstract

**Background:**

In head and neck squamous cell carcinoma (HNSCC), solute carrier family 16 member 1 (SLC16A1) is associated with tumor advancement and reduced sensitivity to ferroptosis, yet the molecular basis of these effects remains unclear. This study seeks to uncover how SLC16A1 contributes to HNSCC tumorigenesis.

**Methods:**

To elucidate how SLC16A1 drives HNSCC progression via ferroptosis resistance, we performed RNA sequencing on SLC16A1-knockdown HNSCC cells and controls, followed by functional validation. We next systematically assessed the role of the candidate molecule solute carrier family 7 member 11 (SLC7A11) in HNSCC progression and resistance to ferroptosis using loss- and gain-of-function experiments *in vitro* and xenograft-based assays *in vivo*. Finally, we applied RNA interference and validated expression changes by quantitative real-time polymerase chain reaction and immunoblotting to map the signaling pathway by which SLC16A1 controls SLC7A11 expression.

**Results:**

Integrated RNA sequencing and functional assays identified SLC7A11 as a key downstream effector of SLC16A1. SLC7A11 mediates SLC16A1-driven tumor cell proliferation, ferroptosis resistance, and tumorigenesis. Mechanistically, SLC16A1 activates signal transducer and activator of transcription 3 (STAT3) to transcriptionally upregulate SLC7A11 expression.

**Conclusion:**

Our study defines a novel SLC16A1–STAT3–SLC7A11 signaling axis that promotes HNSCC progression by conferring robust resistance to ferroptosis. This axis may be leveraged as a therapeutic target to mitigate treatment resistance.

## 1 Introduction

Head and neck squamous cell carcinoma (HNSCC) is a global top-10 cancer by incidence and poses a substantial burden of cancer-related morbidity and mortality [1,2]. With an estimated 900,000 new cases annually, it represents a major global public health challenge [2]. Histologically, HNSCC arises from squamous mucosa in upper head-and-neck mucosal sites, most commonly in the mouth, throat (oropharynx), and laryngeal region [3]. Clinically, this malignancy is marked by aggressive local invasion and early metastasis to cervical lymph nodes [4]. Established risk determinants for HNSCC include tobacco use, alcohol abuse, high-risk HPV carriage (notably HPV16), and long-standing laryngopharyngeal reflux [5–8]. Due to the relatively concealed anatomical locations of these cancers, a substantial proportion of HNSCC cases are identified only after the disease has progressed to locoregionally advanced stages, which significantly limits treatment efficacy and worsens prognosis [8]. Despite notable advancements in diagnostic techniques and therapeutic approaches over recent years—including surgical resection, radiotherapy, chemotherapy [9], targeted therapy (such as anti-EGFR drugs like cetuximab [10]), and immunotherapy (PD-1 inhibitors such as nivolumab [11] and pembrolizumab [10,12])—the prognosis for HNSCC remains poor, with five-year survival rates stagnating at approximately 50% over the past three decades [13,14]. Given these challenges, it is crucial to delve deeper into the pathogenesis and molecular basis of HNSCC. Identifying new biomarkers and exploring effective therapeutic targets are essential for improving patient outcomes. In this context, our preliminary data suggest that solute carrier family 16 member 1 (SLC16A1) promotes oncogenic processes in HNSCC; however, the exact mechanisms remain to be clarified [15].

Ferroptosis denotes an iron-dependent regulated cell death program that results from the accumulation of lipid peroxides [16]. Lipid peroxidation is a process in which cellular lipids undergo oxidative degradation, yielding lipid hydroperoxides as the primary reactive intermediates. These hydroperoxides can further decompose into secondary breakdown products, among which malondialdehyde (MDA) is widely regarded as the most mutagenic [17]. Glutathione peroxidase 4 (GPX4), utilizing reduced glutathione (GSH) as a cofactor, suppresses ferroptosis by detoxifying lipid hydroperoxide [18]. As the functional subunit of system xc^−^, SLC7A11 mediates cystine uptake and contributes to GSH biosynthesis, thereby preserving GPX4 activity and restraining lipid peroxidation to block ferroptosis [19,20]. Erastin and RSL3 trigger ferroptosis by targeting SLC7A11 and GPX4, respectively. In contrast, liproxstatin-1 (Lip-1) acts as a specific inhibitor that suppresses lipid peroxidation and rescue cells from ferroptotic death [21]. Accumulating evidence highlights the critical involvement of ferroptosis in a range of pathological conditions, such as cancer, neurodegenerative disorders, cardiovascular diseases, and respiratory illnesses [22–24]. In cancer therapy, selectively inducing ferroptosis exploits the inherent oxidative stress vulnerabilities of tumor cells, offering a potent and targeted anticancer approach [25]. Therefore, elucidating the molecular regulation of ferroptosis—especially its role and associated signaling networks in HNSCC—not only helps uncover novel mechanisms underlying tumor development and progression but also provides important theoretical foundations and potential intervention targets for developing innovative anti-cancer therapies.

SLC16A1 is a proton-coupled monocarboxylate transporter that mediates the bidirectional exchange of lactate, pyruvate, and ketone bodies across the plasma membrane [26,27]. Under normal physiological conditions, SLC16A1 is crucial for energy metabolism by facilitating lactate transport—generated by skeletal muscle during intense exercise—to the heart or liver, where it is used for oxidative metabolism or gluconeogenesis [28]. It also contributes to the inter-organ distribution and metabolic utilization of ketone bodies. Studies have demonstrated that SLC16A1 is essential for embryonic development and metabolic homeostasis: complete knockout of this gene in mice results in embryonic lethality, whereas heterozygous deletion (Slc16a1+/−) confers resistance to diet-induced obesity [29]. SLC16A1 function is profoundly perturbed during tumorigenesis and progression. It is implicated in several oncogenic events, such as promoting proliferative capacity, increasing invasiveness, facilitating vascular formation, reshaping cellular metabolism, and suppressing immune responses [30–34].

Our prior research has demonstrated that SLC16A1 is markedly overexpressed in HNSCC, with high expression levels strongly correlating with tumor progression and adverse clinical outcomes [15]. Nevertheless, the precise mechanisms through which SLC16A1 drives HNSCC progression have yet to be fully defined. Elucidating the oncogenic functions and regulatory network of SLC16A1 in HNSCC may uncover actionable targets within cancer metabolism for therapeutic intervention. This study seeks to uncover how SLC16A1 contributes to HNSCC tumorigenesis.

## 2 Materials and Methods

### 2.1 Reagents and Antibodies

Erastin (HY-15763), S3I-201 (HY-15146), Colivelin (HY-P1061), and AZD3965 (HY-12750) were acquired from Med Chem Express (Monmouth Junction, NJ, USA). Lip-1 (HY-12726), Dimethyl sulfoxide (DMSO) (D8418), and Puromycin (P8230) have been described previously [15]. Primary antibodies against SLC16A1 (Cat#20139-1-AP), SLC7A11 (Cat#ab307601), β-actin (Cat#A1978), Ki-67 (Cat#34330), and p-signal transducer and activator of transcription 3 (STAT3, Tyr705) (Cat#9145S) were obtained from Proteintech (Wuhan, China), Abcam (MA, USA), Sigma-Aldrich (MO, USA), and Cell Signaling Technology (MA, USA), respectively. Beyotime (Shanghai, China) provided the HRP-coupled goat anti-rabbit IgG (A0208) and goat anti-mouse IgG (A0216) antibodies used in this study.

### 2.2 Cell Culture

The human laryngeal squamous cell carcinoma (TU177) (HTX2967; Otwo Biotech, Shenzhen, China) and human tongue squamous cell carcinoma (HN6) (HTX2974; Otwo Biotech) have been described previously [15]. Cells were cultured in DMEM (BC-M-005; Biochannel, Nanjing, China) supplemented with 10% fetal bovine serum (S711-001S; Lonsera, Suzhou, China) as well as 1% penicillin–streptomycin (C0222; Beyotime), at 37°C in 5% CO_2_. Mycoplasma testing and STR-based authentication were performed regularly for all cell lines, and experiments were carried out only with mycoplasma-free cultures.

### 2.3 High-Throughput RNA Sequencing (RNA-Seq) Analysis

TU177 cells (SLC16A1 knockdown vs. control) were processed for total RNA preparation with TRIzol (15596026CN; Invitrogen, CA, USA), and three biological replicates were included for each group. Stranded mRNA libraries were prepared using the Illumina TruSeq^TM^ RNA Library Prep Kit (RS-122-2001; Illumina, CA, USA) from RNA samples with RIN ≥ 7.0, verified on an Agilent 2100 Bioanalyzer (Agilent Technologies, CA, USA). Library QC was performed with an Agilent Bioanalyzer, and sequencing was conducted on an Illumina NovaSeq 6000 instrument (Cosmos Wisdom, Hangzhou, China) using a paired-end 2 × 150 bp configuration, generating approximately 30 million reads per sample. Raw read counts were imported into DESeq2, and library size differences were corrected using the median-of-ratios normalization method. Dispersion estimates were obtained by first fitting a gene-wise dispersion model and then shrinking these estimates toward a fitted curve based on the mean-dispersion relationship. Differentially expressed genes (DEGs) were called with DESeq2 based on Wald statistics, with multiple-testing correction performed using the Benjamini–Hochberg procedure. The criteria for significance included an adjusted *p*-value < 0.05 and an effect size of at least 1.5-fold (|log_2_FC| ≥ 0.585). The complete DEGs list is available in Supplementary Table S1. Kyoto Encyclopedia of Genes and Genomes (KEGG) enrichment was carried out with clusterProfiler in R; pathway overrepresentation was evaluated by hypergeometric testing and reported after Benjamini–Hochberg false discovery rate correction, with false discovery rate < 0.05 indicating significance.

### 2.4 Western Blotting

Cells were lysed in RIPA (P0013B; Beyotime) as described previously [15,35], after which the lysates were heated at 100°C for 10 min. Electrophoretic separation was performed using 10% SDS–PAGE (40 μg protein per lane), followed by electrotransfer to PVDF membranes (IPVH00010; Millipore, MA, USA). Membranes were preblocked in 5% non-fat milk at room temperature for 1 h. Primary antibody incubation was then carried out overnight at 4°C using a 1:1000 dilution. Following appropriate washes, binding of HRP-conjugated secondary antibody (1:1000) was carried out for 1 h at room temperature. Chemiluminescent signals were captured using a ChemiScope 6000 imaging system (Clinx Science Instruments, Shanghai, China). ImageJ (v1.53a; NIH, MD, USA) was used for densitometric quantification, with β-actin serving as the loading control for normalization.

### 2.5 Quantitative Real-Time Polymerase Chain Reaction (qRT-PCR)

According to established procedures [15,35], TRIzol reagent (Invitrogen) was used to isolate total RNA from cultured cells. RNA purity, confirmed by OD_260/280_ ratios between 1.8 and 2.0 on a Nano-300 spectrophotometer (Allsheng, Hangzhou, China), ensured suitability for downstream applications. First-strand cDNA was generated from 1 μg total RNA using the SR511 kit (Genesand, Beijing, China). We conducted qRT-PCR in triplicate on the LightCycler^®^ 96 System (Roche, Switzerland) using TB Green^TM^ Premix Ex Taq^TM^ II (RR820Q; Takara, Shiga, Japan). Each qPCR reaction (20 μL total) comprised 10 μL premix, 0.8 μL each of forward and reverse primers (10 μM), 2 μL cDNA template, and nuclease-free water to the final volume. Cycling parameters included an initial 95°C denaturation for 30 s and 40 amplification cycles of 95°C for 5 s plus 60°C for 20 s. A melting curve analysis (65–95°C, 0.1°C/s) confirmed single-peak amplification for all primer pairs, indicating target specificity. The 2^−ΔΔCt^ method was used to estimate relative mRNA expression, with β-actin as the internal control and the empty-vector group as the calibrator. Primers were supplied by Sangon Biotech (Shanghai, China); sequences are as follows:

SLC7A11: F: 5^′^-GGTGGTGTGTTTGCTGTC-3^′^, R: 5^′^-GCTGGTAGAGGAGTGTGC-3^′^; β-actin: F: 5^′^-ATCGTCCACCGCAAATGCTTCTA-3^′^, R: AGCCATGCCAATCTCATCTTGTT-3^′^.

### 2.6 Lentivirus Infection

Lentiviral vectors for overexpression and knockdown (both at 1 × 10^8^ TU/mL) were obtained from GenePharma (Shanghai, China). These included the LV4 vector system for overexpression of *SLC16A1* or *SLC7A11* cDNA, as well as the corresponding empty vector, and the LV-2N vector system for RNA interference, expressing gene-specific shRNAs against *SLC16A1* and *SLC7A11*, together with a scrambled shRNA negative control (shSc). The sequences of all shRNAs were detailed in Supplementary Table S2. Lentiviral particles were produced and used to establish stable cell lines as previously described [35]. Target cells were infected with lentiviral vectors (MOI 10–20) plus Polybrene (10 μg/mL), followed by puromycin selection (2 μg/mL) starting at 48 h and lasting 7 days. Knockdown or overexpression was confirmed by Western blot.

### 2.7 RNA Interference

STAT3-specific siRNA oligonucleotides were synthesized by Tsingke Biotechnology (Beijing, China). Transfection was performed at ~70% confluence after seeding 1 × 10^5^ cells into each well of a 12-well plate. siRNA–Lipofectamine complexes were generated by diluting 20 pmol siRNA and 1 μL Lipofectamine^TM^ 2000 (11668500; Thermo Fisher Scientific, MA, USA) in serum-free DMEM (50 μL each) and incubating the mixture for 20 min at room temperature; the complexes were then gently dispensed into wells. Knockdown was validated by Western immunoblotting using lysates prepared after 48 h incubation at 37°C. Results were confirmed across at least three independent siRNA experiments. The target sequences of the STAT3-siRNAs are listed below:

siNC: 5^′^-TTCTCCGAACGTGTCACGT-3^′^;

siSTAT3^1^: 5^′^-CATCTGCCTAGATCGGCTA-3^′^;

siSTAT3^2^: 5^′^-AGTCAGGTTGCTGGTCAAA-3^′^.

### 2.8 Cell Viability, Proliferation, EdU Staining and Colony Formation Assays

Cell viability was assessed using the CCK-8 assay kit (C0037; Beyotime). Cells were seeded into 96-well plates (1 × 10^4^/well), allowed to attach overnight, and then incubated with erastin or 0.1% DMSO for 24 h. After adding 10 μL CCK-8 per well and incubating at 37°C for 1 h, absorbance was measured at 450 nm using a Multiskan^TM^ FC microplate reader (Thermo Fisher Scientific). The IC_50_ was derived from concentration–response curves fitted to the experimental data. Experiments were run with triplicate wells per condition and were validated across three independent experiments using different cell batches.

Cell proliferation was also assessed using the CCK-8 assay kit (C0037, Beyotime). Cells were seeded into 96-well plates at 1 × 10^3^ cells per well and left to attach overnight. Cell viability was assessed at 24, 48, 72, and 96 h after plating by adding 10 μL CCK-8 to each well and incubating at 37°C for 1 h. A Multiskan^TM^ FC microplate reader (Thermo Fisher Scientific) was used to read absorbance at 450 nm. Blank wells containing medium only (no cells) were included on each plate to subtract background absorbance. Data are presented as a growth curve plotted using absorbance values (OD_450_).

In the EdU assay, engineered TU177 cells with stably silenced *SLC16A1* and enforced *SLC7A11* expression, HN6 cells with *SLC16A1* overexpression and *SLC7A11* knockdown, and their respective controls were seeded onto glass-bottom dishes (1.5 cm diameter; 4 × 10^4^ cells/dish) and labeled with EdU for 2 h at 37 °C using the BeyoClick^TM^ EdU-488 Kit (C0071S; Beyotime). After routine fixation and permeabilization, EdU incorporation was detected according to the manufacturer’s instructions. DAPI (5 μg/mL; Beyotime) was applied for nuclear staining at room temperature for 5–10 min in the dark. Samples were then quickly washed with PBS. Image acquisition was performed on a Carl Zeiss LSM880 + 225 Airyscan confocal system. EdU incorporation was quantified by determining the proportion of EdU^+^ cells within the DAPI^+^ nuclear population. Data were obtained from three technical replicates per condition across three independent repeats using separate cell batches.

Under humidified conditions (37°C, 5% CO_2_), 6-cm dishes were cultured for 7 days after plating 1 × 10^3^ cells per dish. Fixation was performed with 4% paraformaldehyde (30 min), and colonies were subsequently visualized by 0.1% crystal violet staining for 30 min. Colony numbers were determined by manual counting or by ImageJ (version 1.53a; National Institutes of Health) based on brightfield images acquired with a BX53M microscope (Olympus, Tokyo, Japan).

### 2.9 Lipid Reactive Oxygen Species (L-ROS) Assay

The detailed protocols for L-ROS have been previously described [35]. Briefly, 1 × 10^6^ cells were seeded into 6-well plates, incubated with erastin or vehicle for 24 h, and subsequently stained using 2 μM C11-BODIPY^581/591^ (D3861; Thermo Fisher Scientific) for 25 min at 37°C in the dark. After PBS rinses, cells were trypsinized, pelleted by centrifugation, and resuspended in 400 μL PBS for dual-channel fluorescence measurement.

### 2.10 MDA Assay

Cells (4 × 10^5^) were seeded into 6-cm dishes and cultured overnight to reach ~80% confluence, followed by 24 h erastin exposure (9 μM for TU177; 5 μM for HN6). MDA content was then quantified using Beyotime’s MDA lipid peroxidation kit (S0131S) according to the manufacturer’s guidelines. Cells were lysed after treatment. Lysates were spun at 12,000× *g* for 10 min at 4°C, and the cleared supernatant was harvested for MDA determination.

Tissue samples were processed on ice in RIPA buffer (P0013B; Beyotime) by homogenization and sonication. The homogenate was centrifuged at 12,000× *g* for 15 min at 4°C, and the cleared supernatant was collected for downstream analyses. Supernatant MDA was quantified using the assay kit guidelines and normalized to total protein (nmol/mg protein). Protein concentration in both cell and tissue lysates was obtained using a BCA kit (P0012S; Beyotime) with a calibration curve.

### 2.11 GSH Assay

GSH was assayed using a published method [35]. Briefly, cells grown in 6-cm dishes were exposed to erastin for 24 h, collected, and resuspended. Protein removal reagent M was added to the cell suspension, followed by vigorous vortexing and two cycles of rapid freeze–thaw. A Multiskan^TM^ FC microplate reader (Thermo Fisher Scientific) was used to measure the 412 nm signal immediately after the supernatant was reacted with detection reagents from the Beyotime GSH/GSSG assay kit (S0053). The GSH/GSSG ratio was calculated from the standard curve using the formula: GSH = total glutathione − 2 × GSSG.

### 2.12 In-Vivo Tumor Models

Twenty-one male BALB/c nude mice (18–22 g, 6 weeks old) were acquired from GemPharmatech (Nanjing, China). After a 1-week acclimation, mice were maintained at 22 ± 2°C in SPF housing with a 12-h light/dark cycle and given chow and water freely. For the *in vivo* tumor xenograft model, the mice were allocated to groups by computer-generated randomization, which was carried out by an investigator who did not participate in subsequent measurements. A total of seven experimental groups were established (n = 3 per group): (1) TU177-shSc + vector (empty vector with scrambled shRNA control); (2) TU177-shSLC16A1 + vector (SLC16A1 knockdown); (3) TU177-shSLC16A1 + SLC7A11-OE (SLC16A1 knockdown with SLC7A11 overexpression); (4) HN6-vector + shSc (empty vector with scrambled shRNA control); (5) HN6-SLC16A1-OE + shSc (SLC16A1 overexpression with scrambled shRNA); (6) HN6-SLC16A1-OE + shSLC7A11^1^ (SLC16A1 overexpression with SLC7A11 knockdown, shRNA^1^); (7) HN6-SLC16A1-OE + shSLC7A11^2^ (SLC16A1 overexpression with SLC7A11 knockdown, shRNA^2^). To establish tumors, each mouse received a subcutaneous axillary injection of 200 μL DMEM containing 5 × 10^6^ cells. At 3-day intervals, tumor length and width were measured and used to compute volume as (length × width^2^)/2 in mm^3^. Body weight and general health status were tracked continuously during the experiment. Mice were humanely euthanized by CO_2_ asphyxiation when tumors reached 1500 mm^3^ or if signs of distress (e.g., ulceration, >20% body weight loss, lethargy) were observed, in accordance with predefined humane endpoints. Ethical permission for the study was granted by the Animal Ethics Committee of Anhui Medical University (LLSC-20251940), and all procedures were performed under the guidelines of the Animal Center of Anhui Medical University.

### 2.13 Immunohistochemistry (IHC) Analysis

Fixation was performed in 10% neutral buffered formalin at room temperature for 24 h, followed by standard tissue processing (dehydration, clearing, and paraffin embedding). Serial 4-μm paraffin sections were prepared and mounted for immunohistochemistry. For epitope unmasking, sections were heated under pressure in 10 mM sodium citrate (pH 6.0) for 3 min at 125°C, followed by passive cooling to room temperature. Sections were first incubated in methanolic 3% H_2_O_2_ for 15 min to eliminate endogenous peroxidase activity. They were then blocked with 5% normal goat serum prepared in PBS for 1 h before antibody incubation. Sections were incubated overnight at 4°C with primary antibodies specific for SLC16A1, SLC7A11, and Ki-67. After three 5-min PBS washes, slides were placed in a humid chamber and treated with HRP-labeled goat anti-rabbit IgG secondary antibody (A0208; Beyotime; 1:50) at 37°C for 30 min. Signal visualization was developed with a DAB substrate kit (P0201S; Beyotime) following the kit protocol.

### 2.14 Venn Diagram Analysis

A Venn diagram was constructed with the online tool (https://www.bic.ac.cn/test/venn/#/). The ferroptosis suppressor gene set was compiled using FerrDb (version 2.0; http://www.zhounan.org/ferrdb/current/), accessed on 02 April 2025. In FerrDb V2, each gene is assigned a “Score”, defined by the database as the number of studies in which the gene was detected as a ferroptosis suppressor. Only genes with a Score ≥ 4 were included in downstream analysis.

### 2.15 Statistical Analysis

Statistical analysis was conducted in Prism 6.0 (GraphPad Software, CA, USA). Two-group differences were tested using an unpaired two-sided *t*-test, and experiments with ≥3 groups were analyzed by one-way ANOVA with Tukey post hoc correction. Results are expressed as mean ± SD, and significance was defined as *p* < 0.05 (*p < 0.05, **p < 0.01, ***p < 0.001, ****p < 0.0001).

## 3 Results

### 3.1 SLC7A11 Is a Downstream Target Gene of SLC16A1

**Figure 1:**
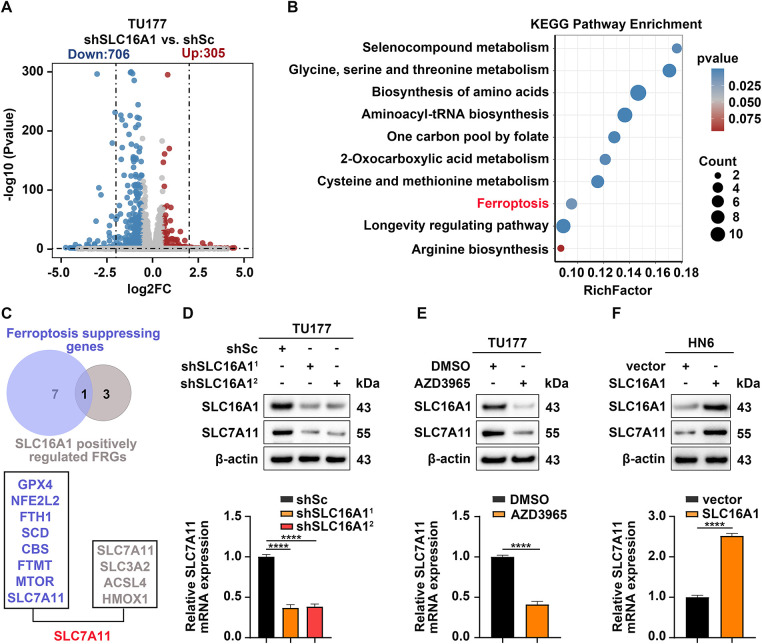
SLC7A11 is a downstream target gene of SLC16A1. (**A**,**B**) RNA-seq of shSLC16A1 vs. shSc TU177 cells. (**A**) DEGs volcano plot. (**B**) KEGG enrichment of downregulated DEGs. (**C**) Intersection of the two datasets. (**D**) TU177 cells with SLC16A1 knockdown and control cells. (**E**) AZD3965 treatment (100 nM, 24 h). (**F**) SLC16A1-overexpressing HN6 and the control cells. (**D**–**F**) SLC7A11 expression levels were analyzed by Western blotting (upper panels) and qRT–PCR (lower panels). Error bars indicate mean ± SD of triplicate samples. ****p < 0.0001.

We previously reported that SLC16A1 accelerates HNSCC initiation and progression and reduces sensitivity to ferroptosis [15]; to further investigate the mechanism by which SLC16A1 confers resistance to ferroptosis, we performed RNA sequencing analysis on SLC16A1-knockdown and control cells. Relative to controls, SLC16A1 knockdown resulted in 1011 differentially expressed genes, comprising 305 upregulated and 706 downregulated transcripts (Fig. 1A). KEGG enrichment analysis of downregulated genes identified ferroptosis as one of the most significantly altered pathways, implicating SLC16A1 in the control of ferroptotic signaling through its influence on core pathway components (Fig. 1B). To identify SLC16A1-driven genes that confer ferroptosis resistance, we overlapped its positively regulated ferroptosis-associated genes with established ferroptosis suppressors. This approach led to the identification of SLC7A11, a critical mediator of ferroptosis that also functions as an oncoprotein in multiple cancer contexts (Fig. 1C). Both qPCR and immunoblotting indicated that SLC16A1 knockdown substantially decreased SLC7A11 mRNA and protein abundance (Fig. 1D). Similarly, treatment of cells with AZD3965, a specific inhibitor of SLC16A1 [36], also resulted in marked decreases in both mRNA and protein levels of SLC7A11 (Fig. 1E). Conversely, SLC16A1 overexpression markedly increased SLC7A11 mRNA and protein abundance (Fig. 1F).

### 3.2 SLC16A1 Promotes Resistance to Ferroptosis by Upregulating SLC7A11

**Figure 2:**
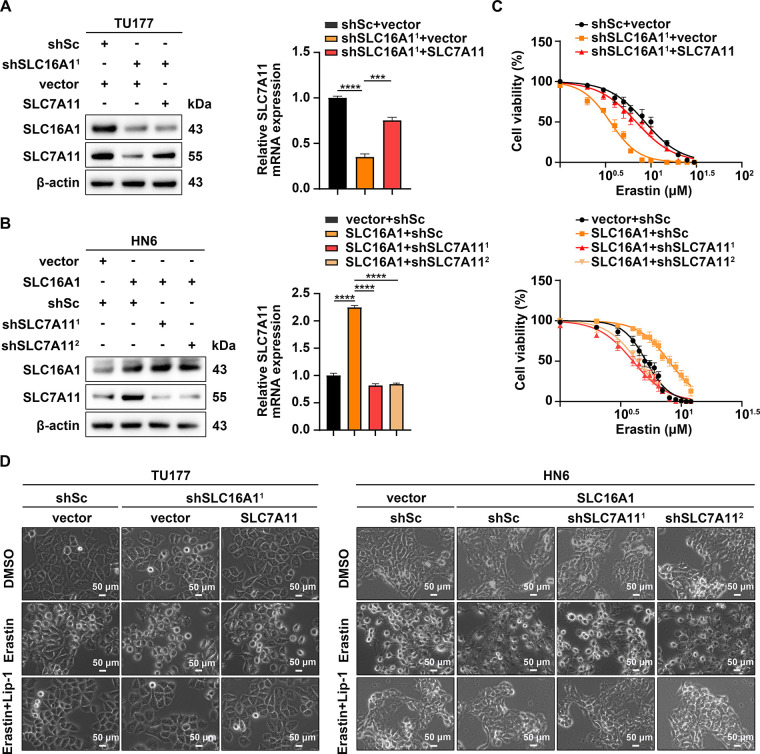
High SLC7A11 levels suppress erastin-induced ferroptosis. (**A**) shSLC16A1 TU177 cells were infected with lentiviruses expressing either an empty vector (vector) or SLC7A11. (**B**) SLC16A1-overexpressing HN6 cells were infected with lentiviruses containing either SLC7A11-targeting shRNAs (shSLC7A11^1^ and shSLC7A11^2^) or a shSc. (**A**,**B**) SLC7A11 expression levels were analyzed by Western blotting (left panels) and qRT–PCR (right panels). (**C**) Cell viability after 24 h of erastin treatment. (**D**) Phase-contrast images of TU177 and HN6 cells treated with erastin (9 μM and 5 μM, respectively) for 24 h in the presence or absence of Lip-1 (1 μM). Scale bar, 50 μm. Error bars indicate mean ± SD of triplicate samples. ***p < 0.001; ****p < 0.0001.

To determine whether SLC16A1 modulates ferroptosis through the regulation of SLC7A11, we conducted complementary experiments. Specifically, SLC7A11 was overexpressed in SLC16A1-knockdown TU177 cells via plasmid transfection (Fig. 2A), whereas endogenous SLC7A11 expression was silenced in SLC16A1-overexpressing HN6 cells using lentivirus-mediated RNA interference (Fig. 2B). Subsequently, to assess ferroptosis sensitivity, cells were exposed to erastin. Our results demonstrated that SLC7A11 overexpression attenuated the heightened sensitivity to erastin-induced ferroptosis in SLC16A1-knockdown TU177 cells (Fig. 2C). Conversely, SLC7A11 silencing exacerbated ferroptotic cell death in SLC16A1-overexpressing HN6 cells under erastin treatment (Fig. 2C). Morphological changes under phase-contrast microscopy further supported these findings. The ferroptosis inhibitor Lip-1 significantly attenuated erastin-induced cell death, demonstrating that ferroptosis underlies this cytotoxic response (Fig. 2D).

**Figure 3:**
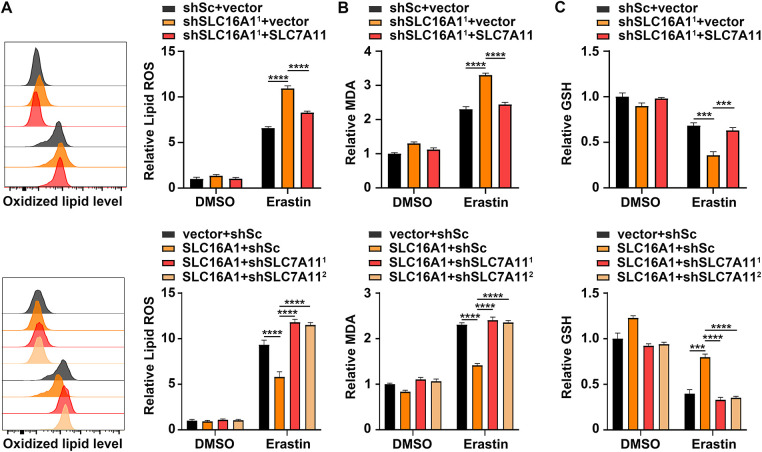
SLC16A1 promotes resistance to ferroptosis by upregulating SLC7A11. (**A**–**C**) TU177 and HN6 cells were treated with erastin (9 μM for TU177; 5 μM for HN6) or vehicle control for 24 h, followed by quantification of L-ROS (**A**), MDA (**B**), and GSH (**C**). Error bars indicate mean ± SD of triplicate samples. ***p < 0.001; ****p < 0.0001.

To investigate the role of SLC7A11 in SLC16A1-driven resistance to ferroptosis, we evaluated key ferroptosis-related biomarkers. Overexpression of SLC7A11 in SLC16A1-knockdown TU177 cells significantly attenuated erastin-induced lipid peroxidation and GSH depletion, indicating restored ferroptosis resistance (Fig. 3A–C). In contrast, SLC7A11 knockdown in SLC16A1-overexpressing HN6 cells reversed the protective effects of SLC16A1 overexpression, leading to increased lipid peroxidation and GSH depletion upon erastin treatment (Fig. 3A–C).

### 3.3 SLC16A1 Promotes HNSCC Cell Proliferation and Tumor Growth by Upregulating SLC7A11

**Figure 4:**
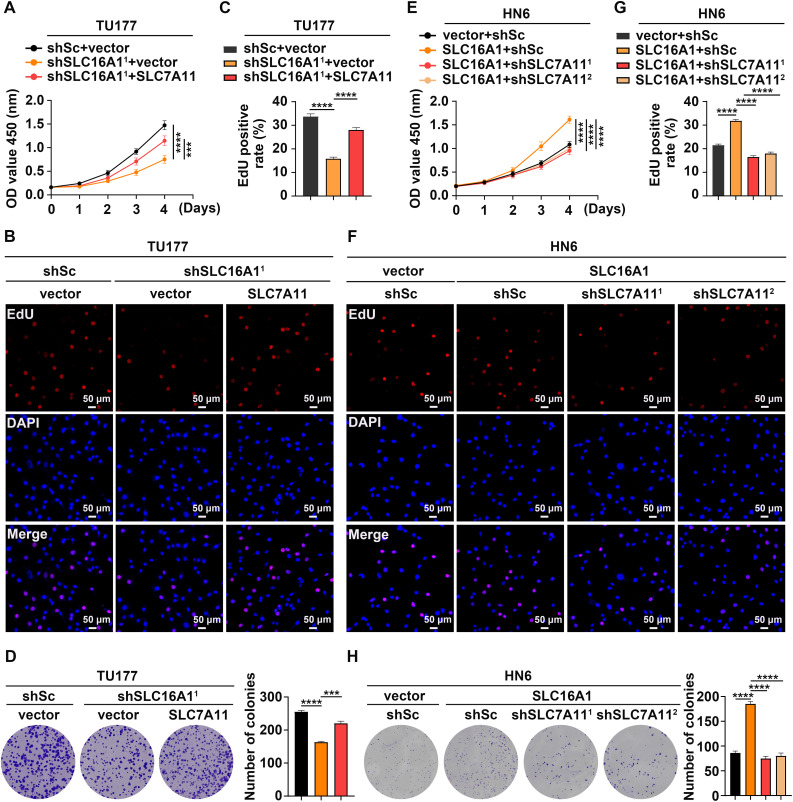
SLC16A1 drives HNSCC cell proliferation via upregulation of SLC7A11. (**A**–**D**) shSLC16A1 TU177 cells transduced with lentivirus expressing SLC7A11 or empty vector control. (**E**–**H**) SLC16A1-overexpressing HN6 cells transduced with lentiviral shRNAs against SLC7A11 (shSLC7A11^1^ and shSLC7A11^2^) or a shSc. Cell proliferation was evaluated by CCK-8 assay (**A**,**E**), EdU assay (**B**,**C**,**F**,**G**), and colony formation assay (**D**,**H**). Scale bar, 50 μm. Error bars indicate mean ± SD of triplicate samples. ***p < 0.001; ****p < 0.0001.

Our prior work established that SLC16A1 drives HNSCC proliferation. To further investigate whether SLC16A1 regulates HNSCC progression through SLC7A11, we conducted a series of functional experiments. In SLC16A1-knockdown cells, SLC7A11 overexpression partially restored proliferation, as evidenced by attenuated growth suppression (Fig. 4A), increased EdU incorporation, and enhanced colony formation (Fig. 4B–D). Conversely, silencing SLC7A11 in SLC16A1-overexpressing HN6 cells markedly blunted the proliferative advantage conferred by SLC16A1 (Fig. 4E–H).

**Figure 5:**
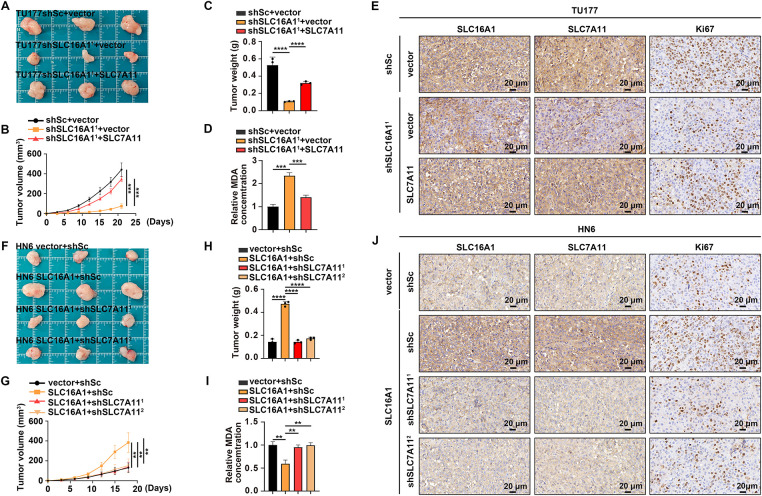
SLC16A1 enhances *in vivo* tumor growth of HNSCC by upregulating SLC7A11. (**A**–**J**) Subcutaneous xenograft tumors generated from the indicated cell lines (n = 3 per group). (**A**,**F**) Representative tumor images. (**B**,**G**) Tumor volumes over time. (**C**,**H**) Tumor weights at endpoint. (**D**,**I**) Relative MDA levels. (**E**,**J**) IHC for SLC16A1, SLC7A11, and Ki-67. Scale bar, 20 μm. Error bars indicate mean ± SD of triplicate samples. **p < 0.01; ***p < 0.001; ****p < 0.0001.

To extend our *in vitro* findings, we evaluated the SLC16A1–SLC7A11 axis in a subcutaneous xenograft model. SLC7A11 restoration in SLC16A1-deficient cells rescued tumor growth, increased Ki-67 staining, and suppressed lipid peroxidation accompanied by decreased MDA (Fig. 5A–E). Conversely, SLC16A1 overexpression significantly enhanced tumor growth and conferred resistance to ferroptosis *in vivo*, whereas these effects were markedly diminished upon SLC7A11 inhibition (Fig. 5F–J). Taken together, our findings suggest that SLC16A1 promotes HNSCC development and progression, at least in part, by upregulating SLC7A11 expression and thereby enhancing resistance to ferroptosis.

### 3.4 SLC16A1 Upregulates SLC7A11 via STAT3 Activation

**Figure 6:**
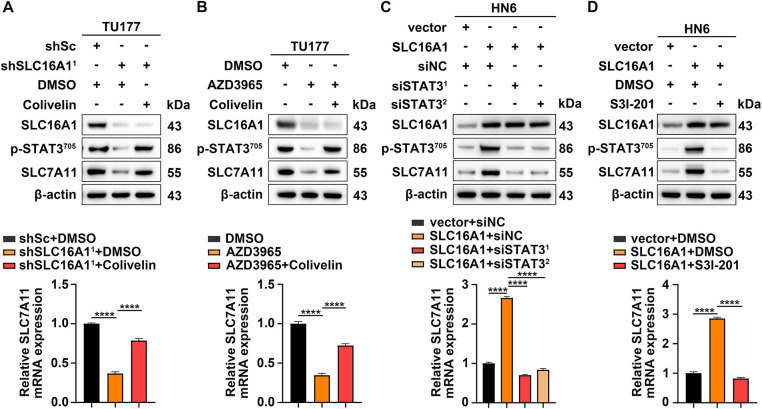
SLC16A1 upregulates SLC7A11 via STAT3 activation. (**A**) shSLC16A1^1^ TU177 cells were treated with or without colivelin (1 μM) for 24 h. (**B**) TU177 cells pretreated with AZD3965 and subsequently exposed to colivelin for 24 h. (**C**) SLC16A1 overexpressing HN6 cells transfected with STAT3-targeting or non-targeting siRNAs (siNC) for 48 h. (**D**) SLC16A1 overexpressing HN6 cells treated with or without S3I-201 (50 μM) for 24 h. (**A**–**D**) SLC7A11 expression was analyzed by Western blotting (upper panels) and qRT–PCR (lower panels). Error bars indicate mean ± SD of triplicate samples. ****p < 0.0001.

STAT3 is an established transcription factor that governs SLC7A11 expression [35,37,38]. These observations led us to test whether SLC16A1 upregulates SLC7A11 through STAT3 activation. To validate this hypothesis, we targeted SLC16A1 using both genetic and pharmacological strategies, which concurrently attenuated STAT3 signaling and reduced SLC7A11 expression (Fig. 6A,B). Importantly, treatment with colivelin, a specific activator of STAT3, effectively restored SLC7A11 expression under these conditions (Fig. 6A,B). In contrast, SLC16A1 overexpression in HN6 cells enhanced STAT3 signaling and upregulated SLC7A11 (Fig. 6C,D). Notably, silencing or pharmacological inhibition (S3I-201, a specific inhibitor of STAT3) [35] of STAT3 resulted in a significant downregulation of SLC7A11 expression (Fig. 6C,D). Collectively, these findings establish that SLC16A1 upregulates SLC7A11 through STAT3 signaling, revealing a previously uncharacterized regulatory axis.

## 4 Discussion

SLC16A1 is commonly upregulated in human cancers and drives tumor progression by rewiring cellular metabolism, acidifying the extracellular milieu, and facilitating immune escape [39–43]. Our previous studies revealed increased SLC16A1 expression in HNSCC; elevated SLC16A1 reinforced ferroptosis resistance and consequently promoted tumor cell proliferation, migration, and invasion [15]. However, the underlying molecular mechanisms through which SLC16A1 mediates this critical biological effect remain poorly understood. In particular, whether SLC16A1 regulates ferroptosis by modulating specific downstream target genes remains an unresolved question. To elucidate the mechanistic basis of SLC16A1 function, we performed transcriptome-wide RNA sequencing analysis on SLC16A1-knockdown HNSCC cells and their corresponding control cells. To specifically identify potential downstream effectors involved in ferroptosis regulation, we conducted a Venn diagram analysis to intersect SLC16A1 positively regulated differentially expressed genes with a curated gene set associated with ferroptosis suppression. This integrative screening strategy enabled the identification of SLC7A11 as a key candidate target. Subsequently, using a combination of genetic approaches—including shRNA-mediated knockdown and plasmid-driven overexpression—and pharmacological interventions—such as treatment with the SLC16A1-specific inhibitor AZD3965—we demonstrated that modulation of SLC16A1 expression or activity consistently altered SLC7A11 levels. Overall, the results support that SLC7A11 functions downstream of SLC16A1 as an essential effector.

SLC7A11 suppresses ferroptosis by importing cystine to support glutathione synthesis, thereby restraining lipid peroxidation and promoting cancer cell survival [44–47]. Therefore, elucidating the regulatory network governing SLC7A11 expression holds significant biological importance and provides a theoretical foundation for ferroptosis-targeted therapeutic strategies. SLC7A11 expression is known to be tightly regulated at multiple levels: transcriptionally, by key transcription factors such as NRF2, ATF4, and p53 [44,48–50]; post-transcriptionally, by RNA-binding proteins (e.g., RBMS1 [50], IGF2BP1 [51]) and non-coding RNAs (e.g., miR-5096 [52]); and post-translationally, through modulation of protein stability via the ubiquitin-proteasome pathway [53–57]. In this study, we identify a previously unknown regulatory axis: SLC16A1–STAT3–SLC7A11. We demonstrate that SLC16A1 activates the STAT3 signaling pathway, promoting its phosphorylation and nuclear translocation, and that active STAT3 engages promoter cis-elements of SLC7A11 to mediate its transcriptional activation. This finding not only establishes, for the first time, a functional link between SLC16A1 and the transcriptional regulation of SLC7A11—defining SLC7A11 as a novel downstream target of SLC16A1—but also validates and extends previous evidence that STAT3 acts as a direct transcriptional activator of SLC7A11 [37,38]. In summary, SLC16A1 confers ferroptosis resistance by elevating SLC7A11 expression through a STAT3-dependent mechanism.

Lactate, a key end product of aerobic glycolysis in cancer cells, functions not merely as a metabolic waste but as a dynamic signaling molecule that actively remodels the tumor microenvironment and drives multiple aspects of tumor progression [58–61]. As the principal mediator of lactate efflux, SLC16A1 enables efficient lactate export into the tumor microenvironment (TME), fueling its remodeling [62–64]. However, despite the well-documented roles of SLC16A1-mediated lactate transport in tumor metabolism and immune modulation, its involvement in regulating tumor cell sensitivity to ferroptosis—and the potential functional link between lactate transport and ferroptosis resistance—remains to be elucidated. These gaps highlight the need for systematic mechanistic studies to fully elucidate the underlying pathways.

## 5 Conclusions

Overall, this study not only uncovers the critical role of the SLC16A1–STAT3–SLC7A11 signaling axis in HNSCC and broadens the current understanding of the SLC7A11 regulatory network, but also establishes a novel theoretical basis for therapeutic strategies aimed at targeting this pathway to modulate ferroptosis and overcome treatment resistance. Future combinatorial approaches—such as SLC16A1 blockade together with ferroptosis-inducing agents—could represent an effective avenue to improve treatment outcomes in HNSCC.

## Supplementary Materials



## Data Availability

The data supporting the findings of this study are available in the article and its Supplementary Materials.

## Supplementary Materials

The supplementary material is available online at https://www.techscience.com/doi/10.32604/or.2026.077171/s1.
