# Supplementary material for: SLC16A1 Activates the STAT3/SLC7A11 Pathway to Mediate Ferroptosis Resistance and Tumor Progression in Head and Neck Squamous Cell Carcinoma
Source: Oncol Res. 2026 Apr 22;34(5):34. doi: 10.32604/or.2026.077171 (PMC13126576; doi:10.32604/or.2026.077171)

# 细胞遗传质量鉴定检测

# Cell Line Authentication Service

---

## STR 基因型检测报告

## 样 品 信 息

**样品编号：**

| 客户样本编号 | 公司编号 |
| --- | --- |
| WSU-HN6 | 20240305-02 |

**样品数量：** 1

**样品性状：** 细胞系

**检测项目：** STR

**送检单位：** **富衡**

**检测方法：** 用 Axygen 的基因组抽提试剂盒提取 DNA，采用 21- STR 扩增方案扩增，在 ABI 3730XL 型遗传分析仪上对 STR 位点和性别基因 Amelogenin 进行检测。

# 检 测 结 果

## (一) 检验基本情况

| 公司编号 | 多等位基因 | 匹配细胞系 | 细胞库 | EV 值 | 匹配说明 |
|---|---|---|---|---|---|
| 20240305-02 | 无 | WSU-HN6 | EXPASY | 1.0 | 完全匹配 |

样本基因型检验结果

- 多等位基因指三等位及以上基因现象。
- 本次检测各细胞分型结果良好。

## (二) 各样本描述

- 20240305-02：该株细胞 DNA 分型在细胞系检索中找到**完全匹配**的细胞系，EXPASY 数据库显示细胞名为 **WSU-HN6**，细胞号对应 **CVCL_5516**。本次检测在该细胞系中**没有发现多等位基因**。

| EV | Cell No. | Cell name | Locus names | | | | | | | | |
|---|---|---|---|---|---|---|---|---|---|---|---|
| | | | D5S818 | D13S317 | D7S820 | D16S539 | VWA | TH01 | AM | TPOX | CSF1PO |
| | *Query (Your Cell)* | | 11,11 | 13,13 | 10,11 | 10,12 | 18,19 | 6,9 | X,Y | 8,10 | 11,11 |
| 1.0(36/36) | CVCL_5516 | WSU-HN6 | ['11', '11'] | ['13', '13'] | ['10', '11'] | ['10', '12'] | ['18', '19'] | ['6', '9'] | ['X', 'Y'] | ['8', '10'] | ['11', '11'] |

- **备注：**待测细胞系与收录于 ATCC, DSMZ（DSMZ 收录了来自 ATCC、 DSMZ、JCRB 和 RIKEN 等 2490 株细胞的 STR 数据）, ExPASy 细胞库（ExPASy 收录了来自于 ATCC、DSMZ、JCRB、ECACC 和 Riken 等数据库约 8,000 株人源细胞 STR 数据）中的 STR 数据匹配，未收录于上述细胞库的细胞将无法匹配。根据 ATCC 标准委员会鉴定标准（ANSI/ATCC ASN-0002-2022）,匹配度 EV≥80%认为它们具有相关性，可能衍生于共同的祖先细胞；匹配度 55%-80%之间，需要结合其它方法进一步的鉴定认证其相关性。

（三）**样本分型结果**

| 细胞的 STR 位点和 Amelogenin 位点的基因分型结果 | | | | | | |
|---|---|---|---|---|---|---|
| Loci | 送检细胞 STR 信息 | | | 细胞库细胞 STR 信息 | | |
| | 送检细胞名：WSU-HN6 | | | 细胞库细胞名：WSU-HN6 | | |
| | Allele1 | Allele2 | Allele3 | Allele1 | Allele2 | Allele3 |
| D5S818 | 11 | 11 | | 11 | 11 | |
| D13S317 | 13 | 13 | | 13 | 13 | |
| D7S820 | 10 | 11 | | 10 | 11 | |
| D16S539 | 10 | 12 | | 10 | 12 | |
| VWA | 18 | 19 | | 18 | 19 | |
| TH01 | 6 | 9 | | 6 | 9 | |
| AMEL | X | Y | | X | Y | |
| TPOX | 8 | 10 | | 8 | 10 | |
| CSF1PO | 11 | 11 | | 11 | 11 | |
| D12S391 | 18 | 19 | | | | |
| FGA | 21 | 25 | | | | |
| D2S1338 | 17 | 25 | | | | |
| D21S11 | 32.2 | 33.2 | | | | |
| D18S51 | 13 | 13 | | | | |
| D8S1179 | 13 | 14 | | | | |
| D3S1358 | 18 | 18 | | | | |
| D6S1043 | 11 | 17 | | | | |
| PENTAE | 8 | 14 | | | | |
| D19S433 | 14 | 14 | | | | |
| PENTAD | 9 | 9 | | | | |
| D1S1656 | 13 | 13 | | | | |

# 其 他 说 明

## (一) 分型方案及位点分布

| | 方案 1 | 方案 2 | 方案 3 | 方案 4 |
|---|---|---|---|---|
| 1 | D3S1358 | D8S1179 | D19S433 | AMEL |
| 2 | VWA | D21S11 | TH01 | D1S1656 |
| 3 | D7S820 | D16S539 | D13S317 | D5S818 |
| 4 | CSF1PO | D2S1338 | TPOX | D12S391 |
| 5 | PENTAE | PENTAD | D18S51 | FGA |
| 6 | | | D6S1043 | |

实验方案及位点

## (二) STR 数据库比对

本公司采用 DSMZ tools 进行细胞系比对，其中包含来自于 ATCC, DSMZ, JCRB 和 RIKEN 数据库的 2455 个细胞系 STR 数据。如果待检测细胞未收录于以上细胞库或这是自行建立的新细胞系将无法进行比对，用户需根据细胞分型结果自行与其他数据库进行比对。

**签发日期：2024-03-11**

| Sample File | Sample Name | Panel | SQO | OS | SQ |
|---|---|---|---|---|---|
| 30_F04_Cellidentification-1-0306.fsa | WSU-HN6 | 21Plex_STR_Panel_v1.2 | | | |

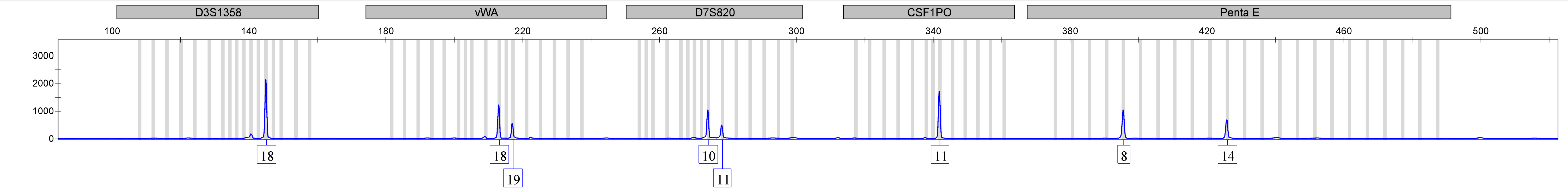


| Sample File | Sample Name | Panel | SQO | OS | SQ |
|---|---|---|---|---|---|
| 30_F04_Cellidentification-1-0306.fsa | WSU-HN6 | 21Plex_STR_Panel_v1.2 | | | |

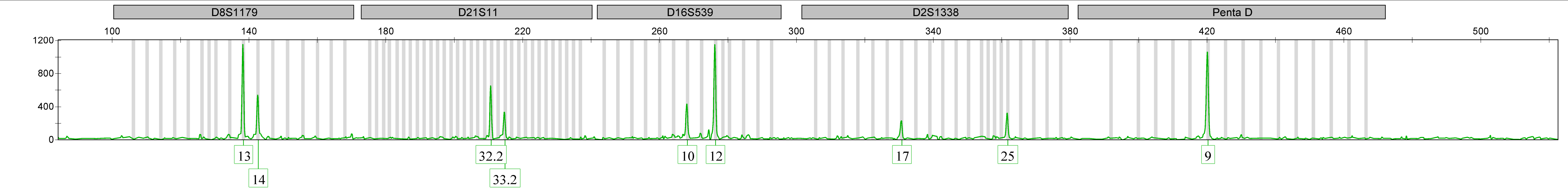


| Sample File | Sample Name | Panel | SQO | OS | SQ |
|---|---|---|---|---|---|
| 30_F04_Cellidentification-1-0306.fsa | WSU-HN6 | 21Plex_STR_Panel_v1.2 | | | |

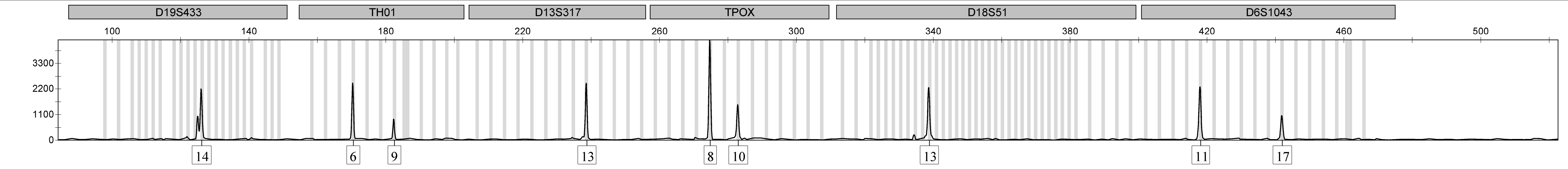


| Sample File | Sample Name | Panel | SQO | OS | SQ |
|---|---|---|---|---|---|
| 30_F04_Cellidentification-1-0306.fsa | WSU-HN6 | 21Plex_STR_Panel_v1.2 | | | |

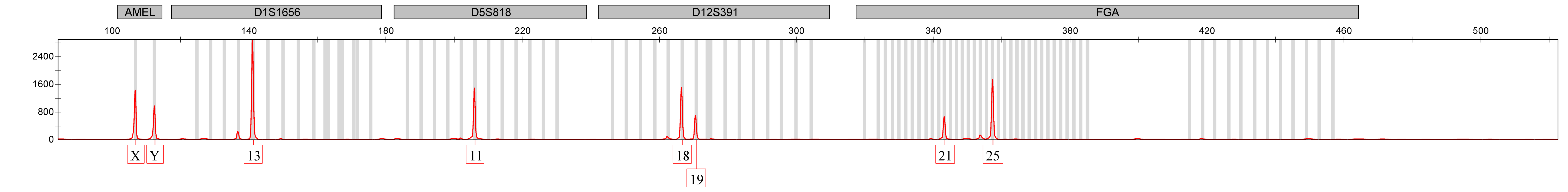

Supplement: Supplementary file 1 [file OncolRes-34-77171-s001.zip › TSP_OR_77171-Supplementary Materials/HN6_STR_Report.pdf.pdf]
