# Supplementary material for: SLC16A1 Activates the STAT3/SLC7A11 Pathway to Mediate Ferroptosis Resistance and Tumor Progression in Head and Neck Squamous Cell Carcinoma
Source: Oncol Res. 2026 Apr 22;34(5):34. doi: 10.32604/or.2026.077171 (PMC13126576; doi:10.32604/or.2026.077171)
Supplement: Supplementary file 1 [file OncolRes-34-77171-s001.zip › TSP_OR_77171-Supplementary Materials/TU177_STR_Report.pdf.pdf]

细胞 STR 检测报告

Cell Line STR Authentication Report

检测报告编号：20240922-04

细胞名称：TU177

送检日期：2024.9.16

样品数量：1

样品类型：细胞沉淀

样品处理：细胞沉淀（>1x10^6cells）用 PureLink™ Genomic DNA Mini Kit（Thermofisher K182001）提取基因组 DNA，使用 PowerPlex®18D 系统（美国 Promega DC1802）试剂盒进行基因扩增，通过 ABI 3730xl 型遗传分析仪对 PCR 产物进行检测。

检测结果:

<1>细胞 DNA 分型结果在 DSMZ 细胞库进行匹配，未找到匹配（EV 值大于 0.8）的细胞。发现多等位基因现象，排除人源细胞交叉污染。

<2>该细胞检测图谱清晰，分型结果良好。

<3>本次检测阴性及阳性对照结果正确。

附表 1：TU177 细胞 STR 分型结果及匹配其细胞库信息

Locus	送检细胞名：TU177			细胞库细胞名：		
D5S818	11	12				
D13S317	14	16				
D7S820	10	11				
D16S539	11	14				
VWA	16	17				
TH01	6	6				
AMEL	X	X				
TPOX	8	10				
CSF1PO	11	12				
D12S391	20	21				
FGA	19	20	24			
D2S1338	21	22				
D21S11	28	29				
D18S51	16	16				
D8S1179	15	16				
D3S1358	14	15				
D6S1043	10	19				
PENTAE	9	13				
D19S433	12	12				
PENTAD	8	9				
D1S1656	11	12	13			

附图 1.细胞基因分型图谱

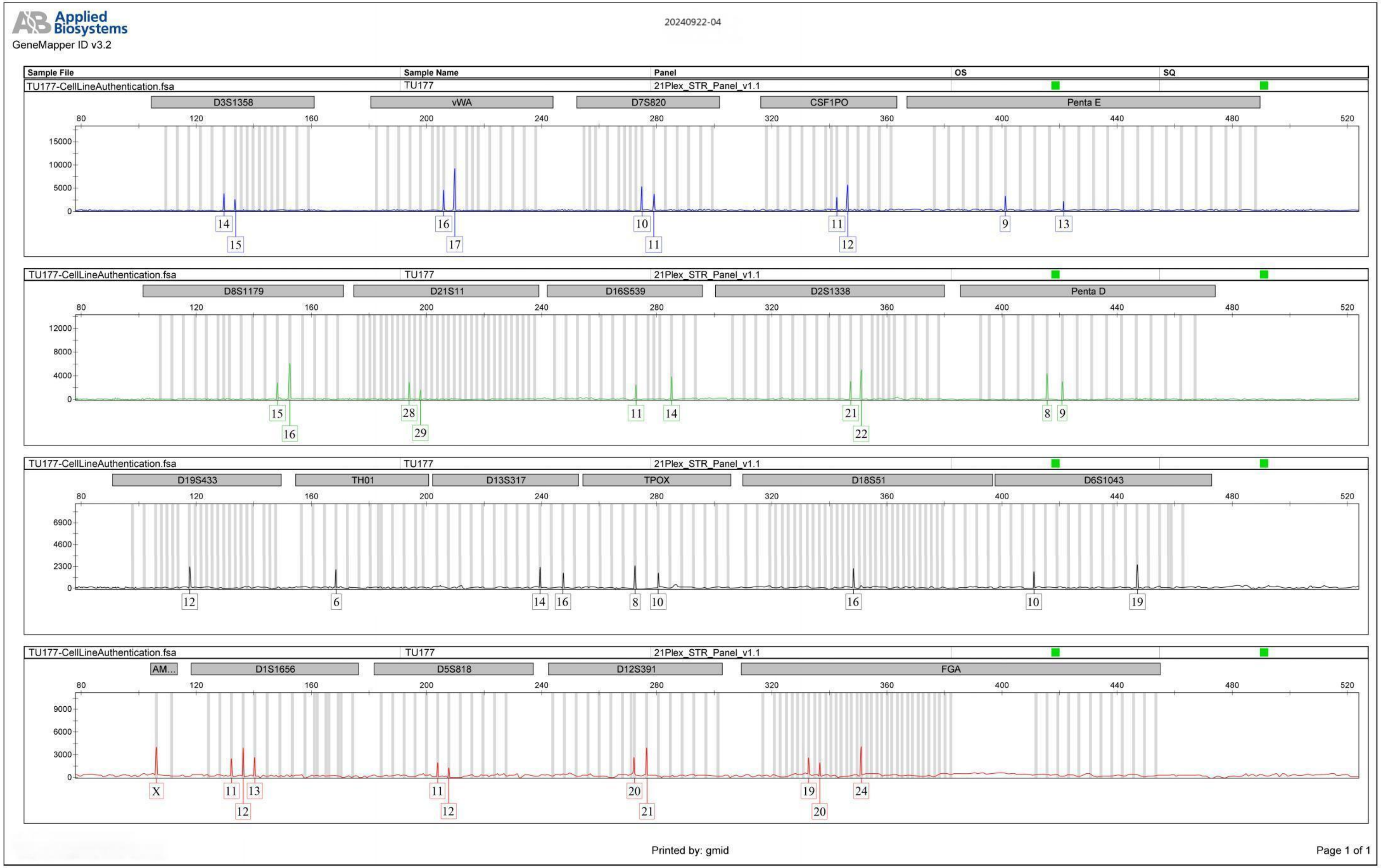

附图 2. STR 位点和 Amelogenin 位点 细胞库匹配结果

Result of STR matching analysis by your data.

- DSMZ Profile Database -

A graphical presentation is shown at the bottom of this page.

EV	Cell No.	Cell name	Locus names									Figures
			D5S818	D13S317	D7S820	D16S539	VWA	TH01	AM	TPOX	CSF1PO	
	Query (Your Cell)		11,12	14,16	10,11	11,14	16,17	6,6	X,X	8,10	11,12	
0.72(26/36)	548	RCH-ACV	11,12	9,14	10,11	11,11	16,17	6,8	X,X	8,11	10,11	-
0.72(26/36)	JCRB1094	SUIT-2	11,12	9,11	9,11	9,9	16,17	6,6	X,X	8,10	11,12	-
0.68(26/38)	362	MOLT-4	11,12	12,13,14	8,10	11,14	17,18,16	6,8	X,Y	8,8	11,12	-
0.67(24/36)	20	BV-173	10,12	8,10	10,11	11,13	16,16	6,9.3	X,X	8,10	11,12	-
0.67(24/36)	CRL-2337	HCC 1937BL	12,12	13,13	9,10	13,14	16,17	6,6	X,X	8,11	11,12	-
0.67(24/36)	CRL-2615	End1/E6E7	11,12	9,12	10,10	10,13	16,17	6,6	X,X	8,11	11,12	-
0.67(24/36)	CRL-7166	Hs 204.Sp	11,11	8,9	10,11	11,13	15,16	6,6	X,X	8,8	11,12	-
0.67(24/36)	CRL-7416	Hs 680.Li	12,12	11,14	9,10	9,11	14,17	6,6	X,X	8,8	11,12	-
0.67(24/36)	CRL-7418	Hs 680.Rec	12,12	11,14	9,10	9,11	14,17	6,6	X,X	8,8	11,12	-
0.67(24/36)	CRL-7419	Hs 680.Sk	12,12	11,14	9,10	9,11	14,17	6,6	X,X	8,8	11,12	-
0.67(24/36)	CRL-7421	Hs 680.Tg	12,12	11,14	9,10	9,11	14,17	6,6	X,X	8,8	11,12	-
0.67(24/36)	CRL-7422	Hs 680.Tr	12,12	11,14	9,10	9,11	14,17	6,6	X,X	8,8	11,12	-
0.67(24/36)	HTB-55	Calu-3	11,11	12,12	10,11	12,14	16,17	6,9.3	X,X	8,8	11,12	-
0.67(24/36)	RCB1724	JHSK-rec	11,12	9,9	10,11	11,13	14,16	6,7	X,X	8,12	11,12	-
0.61(22/36)	48	CRO-AP2	12,12	11,11	10,11	11,11	17,18	6,8	X,X	10,11	11,12	-
0.61(22/36)	124	MONO-MAC-6	11,12	11,12	10,11	12,12	17,17	8,9.3	X,X	8,10	11,12	-
0.61(22/36)	129	CX-1	11,12	11,11	10,10	11,12	17,19	6,9	X,X	8,9	11,12	-
0.61(22/36)	244	CAPAN-1	11,11	9,9	10,11	13,14	16,16	6,6	X,X	8,11	11,11	-
0.61(22/36)	252	MONO-MAC-1	11,12	11,12	10,11	12,12	16,17	8,9.3	X,Y	8,10	11,12	-

STR 匹配结果说明：

1. 根据国际细胞鉴定委员会(ICLAC)制定的细胞STR鉴定标准，细胞系的匹配度≥80% 时，认为它们具有相关性，即衍生于共同的祖先细胞；匹配度在55%至 80% 之间，需要进一步验证相关性；小于55%时，两者不具有相关性。
2. 默认采用DSMZ tools或ATCC tools与细胞库细胞比对，未收录于DSMZ或ATCC等大型细胞库的细胞将无法比对到目的细胞，有其他指定细胞库的请标注细胞库信息。
3. 为了保护细胞及细胞供者隐私，请勿公开所有STR位点信息。如需比对细胞库，提供DSMZ细胞库所需8个位点和性别位点信息即可。
