# Supplementary material for: SLC16A1 Activates the STAT3/SLC7A11 Pathway to Mediate Ferroptosis Resistance and Tumor Progression in Head and Neck Squamous Cell Carcinoma
Source: Oncol Res. 2026 Apr 22;34(5):34. doi: 10.32604/or.2026.077171 (PMC13126576; doi:10.32604/or.2026.077171)
Supplement: Supplementary file 1 [file OncolRes-34-77171-s001.zip › TSP_OR_77171-Table S2.docx]

**Supplementary Table S2. Targeting sequences for shRNẠs used in this study.**

| **Note** | **Sequences (5'-3')** |
| --- | --- |
| shSc | TTCTCCGAACGTGTCACGT |
| shSLC16A1^1^ | CAAAGAGATTGAAGGTATATT |
| shSLC16A1^2^ | ATCAGTCTTCCAAACAATTAA |
| shSLC7A11^1^ | GAGGTCATTACACATATAT |
| shSLC7A11^2^ | TGGAGTTATGCAGCTAATT |
